# New insights in prevention, diagnosis and treatment of stroke: its relation with atrial fibrillation

**DOI:** 10.1007/s12471-012-0263-0

**Published:** 2012-02-28

**Authors:** E. E. van der Wall

**Affiliations:** 1 Interuniversity Cardiology Institute of the Netherlands—Netherlands Heart Instittute, Utrecht, the Netherlands; 2 Department of Cardiology, Leiden University Medical Center, P.O. Box 9600, Leiden, the Netherlands

This special issue of the Netherlands Heart Journal mainly deals with a variety of aspects in patients with atrial fibrillation, including stroke. At the recent American Stroke Association's International Stroke Conference 2012, held in New Orleans, USA from 1 to 3 February, several very interesting presentations around the themes atrial fibrillation and stroke were given. In the first presentation it was reported that the use of outpatient cardiac telemetry over 21 days allowed the detection of occult paroxysmal atrial fibrillation in almost 20% of patients with cryptogenic stroke. Data were obtained from 156 patients (mean age 68.5 years) evaluated by mobile cardiac outpatient telemetry monitoring within 6 months of a cryptogenic stroke or transient ischaemic attack (TIA). It was reported that the detection of paroxysmal atrial fibrillation significantly increased from 3.8% in the initial 48 hours, to 9.2% at 7 days, to 15.1% at 14 days, and to 19.5% by 21 days (*p* < 0.05).

In the second presentation on improving early diagnosis of stroke it was reported that CT angiography (CTA) imaging should be included with brain CT (dual imaging) for early detection of severe arterial stenosis in patients presenting with TIA or minor stroke. It was found that of 491 patients with TIA or minor stroke, the median time to a recurrent stroke was one day, but the median time from stroke onset to CTA imaging was 5.5 hours versus 17.5 hours for diffusion-weighted magnetic resonance imaging.

The next presentation, at the diagnostic level, showed that an analysis of postmenopausal women identified novel lipid and lipoprotein biomarkers associated with the risk of ischaemic stroke. Baseline triglycerides, very-low-density lipoprotein size, and intermediate-density lipoprotein particle number were associated with an increased risk of ischaemic stroke. This suggests that new therapeutics targeting these lipids and lipoproteins might confer better risk protection.

At the therapeutic level, it was reported that warfarin was statistically equal to aspirin in reducing death or stroke in heart failure patients, but increased gastrointestinal bleeds. It was found that of the 2305 patients randomised to warfarin or aspirin, warfarin conferred a non-significant 7% reduced risk of the primary outcome of death or stroke at a mean follow-up of 6 years.

Another presentation, at the therapeutic level, showed that combining clopidogrel (Plavix) with aspirin did not prevent recurrent stroke deep in the brain, and even increased the risk of bleeding and death, according to a double-blind, randomised trial that was prematurely discontinued. Patients receiving the dual antiplatelet therapy had a 2.1% risk of major bleeding—which is almost twice the 1.1% risk of those on aspirin plus placebo (*p* < 0.001). It was also demonstrated that the annual risk of all-cause death was greater with the combined therapy, i.e. 2.1% for aspirin plus clopidogrel compared with 1.4% for aspirin plus placebo (*p* = 0.005).

A more positive presentation on therapeutic approaches in stroke showed that dabigatran (Pradaxa) was more cost-effective than warfarin at preventing stroke in atrial fibrillation patients who have had a prior stroke or TIA. Dabigatran provided 0.36 more qualityadjusted life-years (QALY) than warfarin. It was reported that the cost for those additional QALY was $9000, translating into an incremental cost-effectiveness ratio of $25,000.

With respect to atrial fibrillation *per se*, it was shown that older individuals with higher levels of omega-3 fatty acids may face a lower risk of developing atrial fibrillation. The researchers looked at blood samples from approximately 3300 individuals.

In one of the final presentations it was reported that, surprisingly, nearly half (47%) of stroke survivors may have poorly controlled hypertension. The study involved almost 500 adult stroke survivors who participated in a national survey from 1999 to 2004. Therefore, hypertension post-stroke should be carefully watched and treated accordingly.

Based on the increased rate of strokes and heart attacks, the Million Hearts initiative has been launched in the USA. The goal of the Million Hearts initiative is to reduce the rate of heart attacks and strokes by one million events in the USA over the next 5 years. The initiative was rolled out in September 2011 by the US Health and Human Services department, and supported (among others) by the American College of Cardiology.

Such initiatives have also grounded in Europe. At the end of 2010, the European Society of Cardiology (ESC) published new guidelines for management of atrial fibrillation and stroke risk. The Pan-European Stroke Prevention in Atrial Fibrillation Registry—PREFER in AF—enrolled its first patient on 16 January 2012. The European Stroke Organisation (ESO) aims at the development of public policies to reduce the number of stroke-associated deaths and the reduction of the global burden caused by stroke throughout Europe. Lastly, the Stroke Alliance for Europe (SAFE) represents a strong patient voice which will call on European Governments to take the lead in making stroke prevention a health priority. Based on the new insights, these initiatives have become an utmost need.

